# Data set for comparison of cellular dynamics between human *AAVS1* locus-modified and wild-type cells

**DOI:** 10.1016/j.dib.2015.12.053

**Published:** 2016-01-28

**Authors:** Takeomi Mizutani, Hisashi Haga, Kazushige Kawabata

**Affiliations:** Department of Advanced Transdisciplinary Sciences, Faculty of Advanced Life Science, Hokkaido University, North 10 West 8, Kita-ku, Sapporo 060-0810, Japan

**Keywords:** Genome editing, Myosin regulatory light chain, Cell migration, Mean square displacement

## Abstract

This data article describes cellular dynamics, such as migration speed and mobility of the cytoskeletal protein, of wild-type human fibroblast cells and cells with a modified adeno-associated virus integration site 1 (*AAVS1*) locus on human chromosome 19. Insertion of exogenous gene into the *AAVS1* locus has been conducted in recent biological researches. Previously, our data showed that the *AAVS1*-modification changes cellular contractile force (Mizutani et al., 2015 [1]). To assess if this *AAVS1*-modification affects cell migration, we compared cellular migration speed and turnover of cytoskeletal protein in human fibroblasts and fibroblasts with a green fluorescent protein gene knocked-in at the *AAVS1* locus in this data article. Cell nuclei were stained and changes in their position attributable to cell migration were analyzed. Fluorescence recovery was observed after photobleaching for the fluorescent protein-tagged myosin regulatory light chain. Data here are related to the research article “Transgene Integration into the Human *AAVS1* Locus Enhances Myosin II-Dependent Contractile Force by Reducing Expression of Myosin Binding Subunit 85” [1].

**Specifications Table**

**Table:** 

Subject area	*Biology*
More specific subject area	*Cell migration*
Type of data	*Text, image, graph, figure*
How data was acquired	*Phase contrast and wide-field fluorescent (cell nucleus tracking) images were acquired using an inverted microscope (TE2000; NIKON, Tokyo, Japan) equipped with a digital CMOS camera (ORCA-Flash2.8; Hamamatsu Photonics K.K., Shizuoka, Japan). Magnified fluorescent images were acquired using a confocal microscope (C1; NIKON).*
Data format	*Raw and analyzed*
Experimental factors	*AAVS1-modified cells and wild type cells were stained with Hoechst 33342 (cell-permeant nuclear counterstain) or transfected with a Kusabira Orange-tagged myosin regulatory light chain.*
Experimental features	*Cellular migration to the margin was observed, and migratory trajectories were analyzed.*
Data source location	*Hokkaido University, Sapporo, JAPAN*
Data accessibility	*Analyzed datasets are directly provided with this article*

**Value of the data**

• Cell tracking data from wild-type and *AAVS1*-modified cells are available for computer simulation of cell migration.
• Data from fluorescence recovery after photo bleaching for the fluorescent protein-tagged myosin regulatory light chain are applicable for analysis of myosin binding and diffusion coefficient in wild-type and *AAVS1*-modified cells.
• These data may be used as a benchmark of evaluation of the side effect of *AAVS1*-modified cells.

## 1 Data

The data describes the cell migration speed and the dynamical behavior of myosin regulatory light chain (MRLC) of wild-type human fibroblasts (WT cells) and *AAVS1*-modified cells.

## 2 Experimental design, materials and methods

### 2.1 Data acquisition and analysis

Immunofluorescent micrograph and time series of light microscopy data for WT cells and cells with a green fluorescent protein (GFP) gene knocked-in at the *AAVS1* locus (KI cells) were analyzed and compared.

**Fig. 1:**
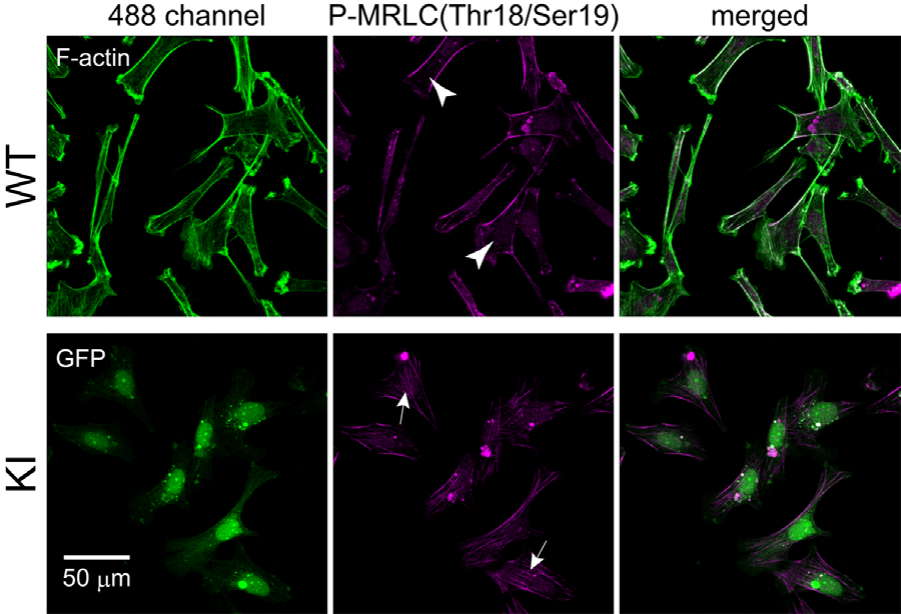
Immunofluorescent micrograph of phosphorylated myosin regulatory light chain in green fluorescent protein gene knocked-in cells (KI) and wild-type cells (WT). KI and WT cells were cultured on a glass substrate, fixed, and stained with phalloidin (F-actin) and anti-phosphorylated MRLC. Scale bar denotes 50 μm.

Representative immunofluorescent images of phosphorylated myosin regulatory light chain (P-MRLC) in WT and KI cells are shown in Fig. 1. KI cells showed both peripheral and interior P-MRLC fibers (Fig. 1 arrows). However, WT cells showed interior P-MRLC fibers (Fig. 1 arrowheads).

**Fig. 2:**
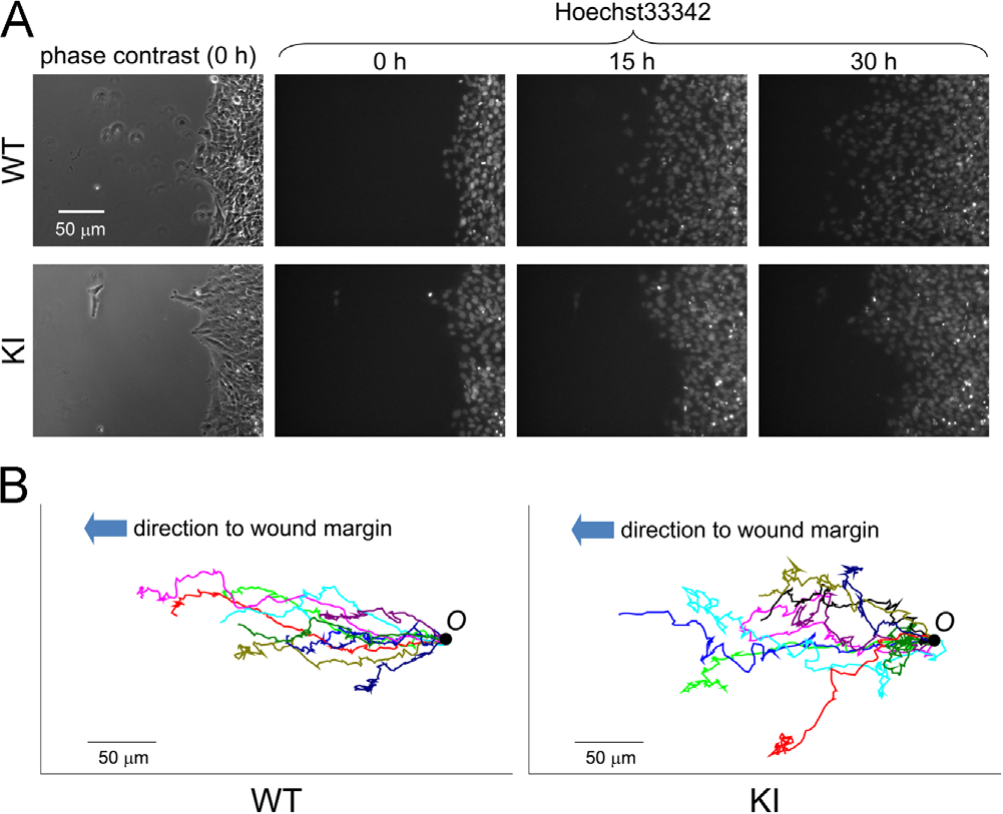
Migration of green fluorescent protein gene knocked-in (KI) and wild-type (WT) cells. (A) Cells were cultured on the glass substrate with a small solid barrier. After a confluent cell monolayer was formed, the solid barrier was removed and cells migrated into the margin. To track cell position, cells were pre-treated with Hoechst 33342 (cell-permeant nuclear counterstain). Fluorescent micrographs of cell nucleus are shown. (B) Representative tracking data from a 30-h observation of ten cells are shown. Scale bars denote 50 μm.

WT and KI cell migration was observed using phase contrast and fluorescent microscopy (Fig. 2). Cells migrated to the margin of the glass substrate (Fig. 2(A) and Appendix A, Appendix A). Representative cell migratory trajectories are shown in Fig. 2(B).

**Fig. 3:**
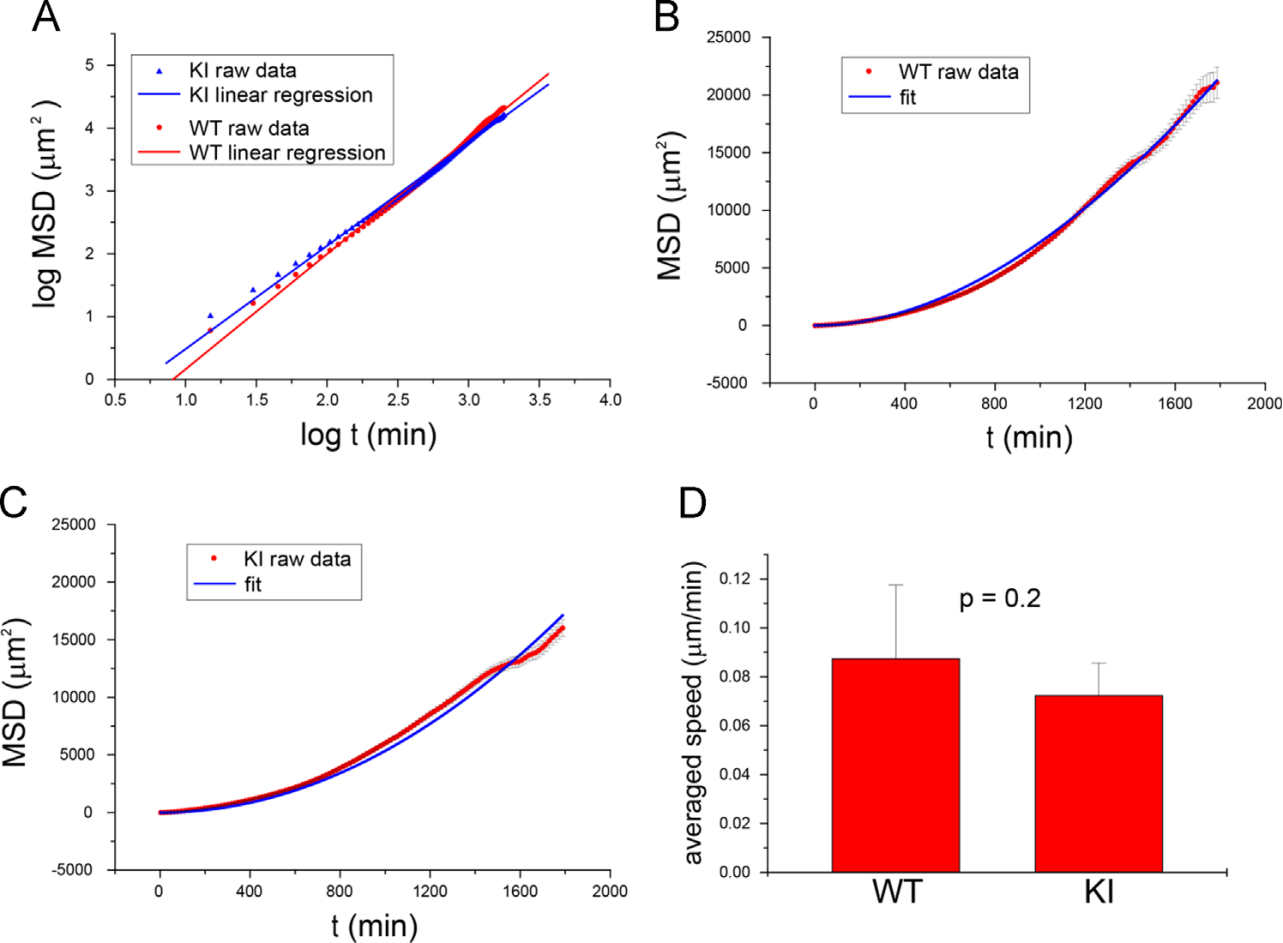
Analysis of mean square displacement (*MSD*) from cell tracking data. *MSD* of WT and KI cells was calculated as a function of the time interval (*t*). (A) *MSD* and *t* are shown in a log-log plot and fitted by least-squares regression. (B, C) *MSD* and *t* are plotted in graphs with linear scaled axes and fitted to the theoretical curves. Cell migration speed was obtained as a fitting parameter. (D) Cell migration speed of WT and KI cells from four independent experiments was averaged and compared by Student’s *t*-test. *P*=0.2. All error bars denote standard error.

We analyzed the mean square displacement (*MSD*; described in the Materials and Methods) from the cell tracking raw data (Supplementary Data 1 and 2) and plotted *MSD* as a function of the time interval (*t*; see Materials and Methods) (Fig. 3). The natural logarithm of *MSD* and *t* was plotted and fitted by least-squares regression to clarify the directionality of cell migration (Fig. 3(A)). For the data set, power indices were 1.8 (WT cells) and 1.6 (KI cells), respectively. These data indicate that the manner of cell migration of each cell type was mono-directional rather than random. To analyze cell migration speed, the *MSD* and *t* data were fit to the theoretical equation of cell migration (see Materials and Methods) (Fig. 3(B) and (C)), and cell migration speed was obtained as one of the fit parameters. Statistical analysis of cell speed from four independent experiments is shown in Fig. 3(D). Although the migration speed of WT and KI cells was not significantly different, as analyzed by Student’s *t*-test (*P*=0.2), WT cells tend to migrated faster than KI cells.

**Fig. 4:**
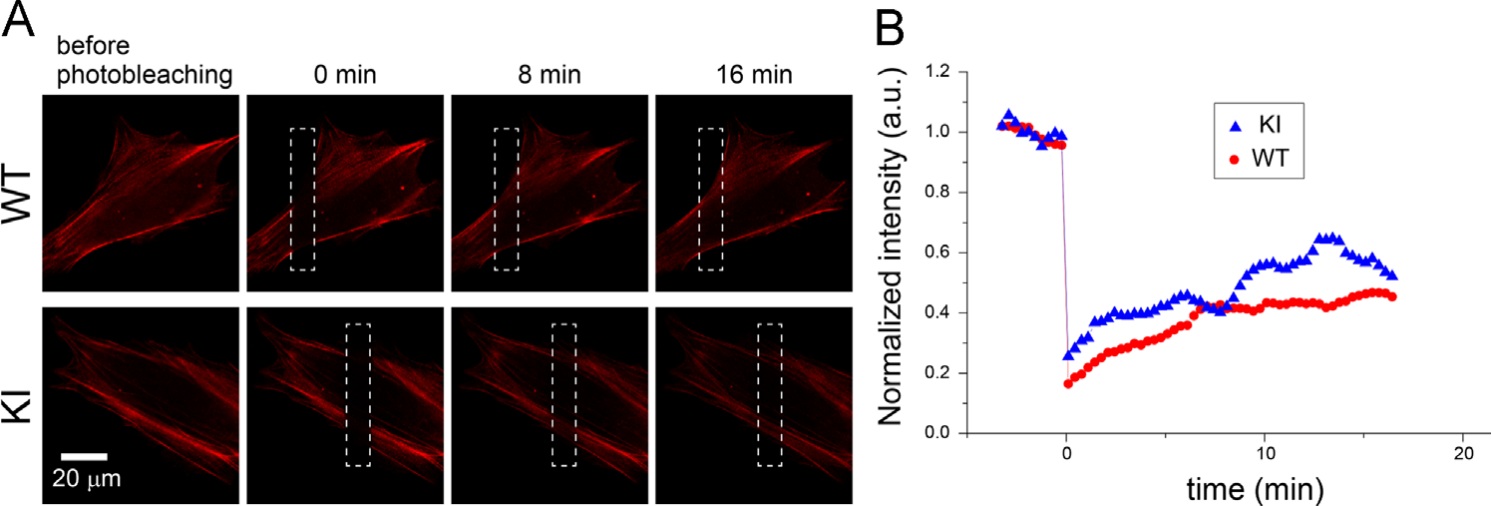
Fluorescence recovery after photobleaching for the fluorescent protein-tagged myosin regulatory light chain (MRLC). Fluorescent protein-tagged MRLC-expressing WT and KI cells were observed using confocal microscope at 20-s time intervals. (A) Representative micrographs before and after local photo bleaching. The photo-bleached regions are indicated as rectangular dashed-line compartments. Scale bar denotes 20 μm. (B) Fluorescent intensity around the photo-bleached regions was averaged and their time course was plotted.

Dynamics of the cytoskeletal protein was observed using confocal microscopy (Fig. 4). Fluorescent protein-tagged myosin regulatory light chain (MRLC)-transfected WT and KI cells were observed before and after local photo bleaching (Fig. 4(A) and Appendix A, Appendix A). Time course of the averaged intensity around the photo-bleached region was plotted (Fig. 4(B) and Appendix A, Appendix A).

### 2.2 Experimental design

Development of KI cells was described in the previous publication [1]. We compared cell migration and dynamics of the cytoskeletal protein in KI and WT cells.

### 2.3 Materials and methods

#### 2.3.1 Cells culture and plasmid construction

The human fibroblast cell line MRC-5 SV1 TG1 was purchased from the RIKEN Cell Bank (Tsukuba, Japan). GFP gene knocked-in cells were established previously [1]. These cells were cultured in low-glucose Dulbecco’s modified Eagle’s medium (DMEM; D6046, Sigma, St. Louis, MO, USA) supplemented with 10% bovine serum and 1% antibiotics (A5955, Life Technologies Corporation, Carlsbad, CA, USA) in 5% CO_2_ at 37 °C. Plasmids were transfected using Lipofectamine 2000 (Life Technologies Corporation).

A plasmid for Kusabira Orange-tagged MRLC expression was constructed as follows. The GFP-coding region of pAcGFP-N3 (Takara Bio Inc., Otsu, Shiga, Japan) was removed by *Bam*HI–*Not*I digestion and replaced with the PCR product from the monomeric Kusabira Orange 2-encoding plasmid (AM-V0141; MBL, Nagoya, Aichi, Japan). The PCR product of wild-type non-muscle MRLC (GenBank accession no. BC004994) was obtained from the MRC-5 cDNA pool and inserted into the *Eco*RI–*Kpn*I site of pKusabiraOrange-N3.

#### 2.3.2 Immunofluorescent microscopy

Cells were fixed with 4% formaldehyde/PBS and permeabilized with 0.5% Triton X-100/PBS. Phosphorylated MRLC was detected using the anti-P-MRLC antibody (#3674; Cell Signaling Technology, Beverly, Massachusetts, USA) and Alexa Fluor-594 rabbit IgG (Life Technologies Corporation). Filamentous actin (F-actin) was stained with Alexa Fluor-488 phalloidin (Life Technologies Corporation). Fluorescence images were obtained with a confocal laser-scanning microscope (C1 confocal imaging system; NIKON, Tokyo, Japan).

#### 2.3.3 Cell migration assay

WT and KI cells were cultured on a glass substrate with a polydimethylsiloxane (PDMS) barrier. After formation of a cell monolayer on the glass substrate, the PDMS barrier was peeled off and cells began to migrate into the empty space [2]. Cell nuclei, stained with Hoechst 33342 (DOJINDO LABORATORIES, Kumamoto, Japan), were used as cell tracking markers. Time-lapse imaging was performed with an inverted microscope (TE2000; NIKON) equipped with a digital CMOS camera (ORCA-Flash2.8; Hamamatsu Photonics K.K., Shizuoka, Japan). Cell tracking was analyzed with the TrackMate plugin in FIJI image analysis software [3]. Data sets for WT and KI cell tracking are attached in the Supplemental data.

Statistical analysis of cell migration was performed follows, as previously reported [4]. Mean square displacement (*MSD*) was calculated as follows:

$$<\mathrm{MSD}\left(t\right)>=<{(x_{i}\left(t_{0}+t\right)-x_{i}\left(t_{0}\right))}^{2}+{(y_{i}\left(t_{0}+t\right)-y_{i}\left(t_{0}\right))}^{2}>,$$

where *x*_*i*_ and *y*_*i*_ denote position of the *i*th cell in a laboratory frame (*x*, *y*), and *t* denotes time interval. In our experimental setup, direction of the cells-to-empty space was taken as –*x*. The *MSD* was used to determine the manner of cell migration by using the following equation [5]:

$$<\mathrm{MSD}\left(t\right)>\propto t^{\alpha }.$$

In case of 1<*α*<2, the cell migration pattern corresponds to anomalous diffusion [5]. The MSD was also used to evaluate cell migration speed by fitting to the following equation [6]:

$$<\mathrm{MSD}\left(t\right)>=2PS^{2}(t-P+Pe^{\frac{-t}{P}}),$$

where *S* denotes average speed and *P* denotes persistence time.

#### 2.3.4 Fluorescence recovery after photo bleaching for the fluorescent protein-tagged MRLC

Kusabira Orange-tagged MRLC-expressing WT and KI cells were cultured on a glass substrate and temporal changes in the fluorescent signal were observed using confocal microscope (C1, NIKON) with a 60× objective lens (CFI-Plan Apo λ series, NIKON). During observation, the scanning area was narrowed to the cell, which forms a photo-bleached spot, and then the scanning area was resized to the original.

## Footnotes

Appendix A Supplementary data associated with this article can be found in the online version at doi:10.1016/j.dib.2015.12.053.
