# Supplementary figures and images for: Data set for comparison of cellular dynamics between human AAVS1 locus-modified and wild-type cells
Source: Data Brief. 2016 Jan 28;6:793–8. doi: 10.1016/j.dib.2015.12.053 (PMC4749938; doi:10.1016/j.dib.2015.12.053)

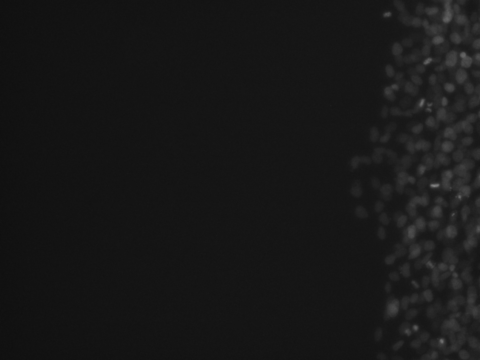

Supplement: Supplementary file 2 — Supplementary material [file mmc2.zip › Supplementary Figure 1.tif]

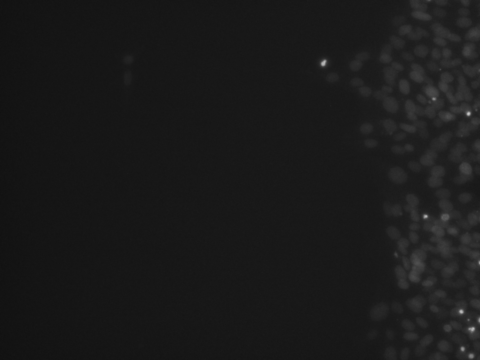

Supplement: Supplementary file 2 — Supplementary material [file mmc2.zip › Supplementary Figure 2.tif]
