# Supplementary material for: Data set for comparison of cellular dynamics between human AAVS1 locus-modified and wild-type cells
Source: Data Brief. 2016 Jan 28;6:793–8. doi: 10.1016/j.dib.2015.12.053 (PMC4749938; doi:10.1016/j.dib.2015.12.053)
Supplement: Supplementary file 2 — Supplementary material [file mmc2.zip › Supplemental information_mizutani.doc]

**Supplemental information of the article entitled as “Data set for comparison of cellular dynamics between human *AAVS1* locus-modified and wild-type cells”**

**Authors:** Takeomi Mizutani, Hisashi Haga, Kazushige Kawabata

**Supplemental Information**

Supplementary Figure 1.tif

Multi-tiff image of migration of Hoechst-stained WT cells. 1 pixel = 1.46 μm. Time interval = 15 (min).

Supplementary Figure 2.tif

Multi-tiff image of migration of Hoechst-stained KI cells. 1 pixel = 1.46 μm. Time interval = 15 (min).

Supplementary Data 1.txt

Nucleus tracking text data of WT cells. Cell tracking was analyzed with the TrackMate plugin in FIJI image analysis software [1]. The tracking results were saved in this text file. “<particle nSpots="*NNN*">” denotes track ID. “detection t="*mmm*"” denotes passage of time. Since the time interval of the cell observation was 15 min, “*mmm”* x 15 min means actual passage of time. “x="uuu" y="vvv"” denotes position of (*X, Y*) in pixel scale. 1 pixel = 0.73 μm.

Supplementary Data 2.txt

Nucleus tracking text data of KI cells. Cell tracking was analyzed with the TrackMate plugin in FIJI image analysis software [1]. The tracking results were saved in this text file. “<particle nSpots="*NNN*">” denotes track ID. “detection t="*mmm*"” denotes passage of time. Since the time interval of the cell observation was 15 min, “*mmm”* x 15 min means actual passage of time. “x="uuu" y="vvv"” denotes position of (*X, Y*) in pixel scale. 1 pixel = 0.73 μm.

Supplementary Figure 3.tif

Multi-tiff image of FRAP experiment for a WT cell. 1 pixel = 0.17 μm. Time interval = 20 (sec). Photo bleaching was performed between the 10^th^ and 11^th^ image.

Supplementary Figure 4.tif

Multi-tiff image of FRAP experiment for a KI cell. 1 pixel = 0.17 μm. Time interval = 20 (sec). Photo bleaching was performed between the 10^th^ and 11^th^ image.

Supplementary Data 3.txt

Time course of normalized fluorescent intensity from the FRAP experiment for a WT cell.

Supplementary Data 4.txt

Time course of normalized fluorescent intensity from the FRAP experiment for a KI cell.

[1] J. Schindelin, I. Arganda-Carreras, E. Frise, V. Kaynig, M. Longair, T. Pietzsch, S. Preibisch, C. Rueden, S. Saalfeld, B. Schmid, J.Y. Tinevez, D.J. White, V. Hartenstein, K. Eliceiri, P. Tomancak, A. Cardona, Fiji: an open-source platform for biological-image analysis, Nature Methods 9 (2012) 676-682.
